# On False Aneurism of the Heart

**Published:** 1829-07-01

**Authors:** 


XLIV.

### FALSE ANEURISM OF THE HEART.*

It is now generally agreed on in the profession to term the dilatation of an artery into a pouch, without any rupture of the coats, a true aneurism; and that accompanied with such rupture, a false one. Most false aneurisms begin by dilatation of the parietes of the vessel, and are, therefore, in their origin true aneurisms, according to the present nomenclature. In proportion as we approach the heart we find that the latter predominate, or at any rate become more frequent; as we recede from the central organ of the circulation, the contrary takes place. The heart itself, especially the left ventricle, is frequently dilated, but seldom presents an example of the false aneurism arising from its walls. Of late years, however, the re-

* *Sur une Espece particuliere d'Aneurisme du Cœur;* par M. Reynaud. Journ. Hebd. No. 22.

searches of Buttner, Baillie, Zannini, Corvisart, the brothers Bérard, Rostan, Cruveilhier, Dance, and Breschet, have put us in possession of many facts of this description. The case of the late General Kyd, published by the Editor of this Journal, was a well-marked, and almost unique case of false aneurism of the heart, bursting into the cavity of the pericardium.

M. Breschet, in his memoir entitled, "Researches and Observations on False Aneurism of the Heart," has arrived at the following conclusions. First, that the heart may be the seat of aneurism, differing from that which it commonly presents. Secondly, that this appears to belong, in an especial manner, to the left ventricle. Thirdly, that the apex of the organ is its ordinary seat. And, fourthly, that this aneurism, in the mode of its formation, the state of its walls, and its contents, appears to come naturally under the denomination of false aneurisms, in the sense ascribed to the term by Scarpa.

In the year 1826, M. Reynaud, the author of the paper we are now to notice, met with an interesting case of the kind at the Hospital of La Charité, in which he was "interne" under M. Lerminier. The patient presented no symptoms of the disease during life. He was about 35 years of age, and sank under formidable nervous symptoms supervening on colica pictonum. The heart presented the following particularities.

The left ventricle was slightly hypertrophied, and its cavity dilated. The internal lining membrane of both the left chambers was opaque, thickened, and of a milky whiteness, in almost the whole of its extent. The mitral valve was thickened towards its free border, especially at the points to which it is attached to the chordæ tendineæ, which themselves were thickened in volume, and of opaque white colour. This latter alteration was particularly noticed in the tendinous chords arising from the columna carnea situate at the posterior part of the ventricle. Beneath this, and for the space of a square inch, the internal membrane was whiter and thicker than at any other part, and separable with ease from a subjacent stratum of thick cellular tissue, which itself could be divided into several layers. The whole texture resembled a good deal that of an artery. Almost in the centre of this altered membrane, and in a point corresponding to the middle of the posterior border of the ventricle, was a rounded opening, capable of receiving the end of the finger, and leading into a nearly spherical cavity. The cavity was large enough to contain a nut, and extended almost to the layer of pericardium immediately investing the heart, from which it was separated only by a very thin layer of muscular fibres. This little aneurismal pouch contained, at its most depending part, a small quantity of fibrinous coagulum, deposited in the form of a membrane on its surface. The walls of the pouch were formed by a dense fibrous membrane, composed of two laminæ, the superficial thin and whitish, the deeper thick, and, like the middle coat of an artery, containing in its substance fibro-cartilaginous and osseous points. This membrane was continuous with the lining membrane of the ventricle, of which it possessed all the characters, save that at the neck of the pouch it was particularly thickened. The muscular fibres of the heart did not actually enter into the formation of the sac, but were separated from it by a layer of cellular tissue, from which it was easy to dissect them, except at that part of the pouch immediately beneath the pericardium verum, where the union was more intimate.

Another and a smaller pouch, entirely filled with old fibrinous coagulum, existed at the middle of the anterior part of the ventricle, near the interventricular septum. Its entrance was narrower than its interior, and the lining membrane of the ventricle prolonged into its interior, which it also lined, was thickened and tumid. The parietes of the pouch were dense, thick, and seemingly fibrous, as in the larger sac described before; its form was spheroidal. Nothing unusual was found in the openings of the heart; the aorta presented some atheromatous depositions in its coats.

It seems that M. Breschet, whose memoir contained the history of ten cases of this description, has come to two general conclusions, one upon the seat of the disease, and the other on the manner in which it takes place. M. Breschet lays down as a proposition, that the apex of the ventricle is the usual spot in which we find the pouch to exist, and probably it is so. M. Reynaud, however, shews in a very satisfactory manner that such is not its constant situation. In three of the cases out of the ten collected by M. Breschet himself, the tumour was obviously in parts of the ventricle distant from its apex; in a heart preserved in the Museum of the Faculty of Medicine of Paris, the superior, anterior, and left side of the ventricle, was the spot affected; in a case recorded by Zannini in his translation of Baillie's Morbid Anatomy, the sac does not appear to have been exactly in the extremity of the ventricle; and, in a specimen at present in the museum of the Chatham Depôt, of which M. Reynaud was informed by "his friend Doctor Carswell," the aneurismal tumour exists at the base of the chamber. Finally, in the case that forms the subject of the author's memoir, both the pouches were in the body and superior part of the ventricle. To these cases we may add that of General Kyd, recorded by Dr. James Johnson, in the sixth volume of this Journal, page 466, in the article on the examination, post mortem, of Talma. In the General's case the aneurism was situated at the base of the ventricle, near the origin of the aorta, and had burst into the pericardium. The absence of any symptoms of disease of the heart in that instance was remarkable. In another case which we shall presently record, the sac was discovered nearly in the same situation. From these observations it is quite clear, that no general rule can be laid down, respecting the exact locality of the sac, and consequently that any theory of the disease founded on such a proposition must necessarily fall to the ground.

So much for the seat of the disease, and now it only remains to consider the mode of its formation. M. Breschet, by the very name he has given the disease, discloses his opinion of its nature. False aneurism implies a sac formed by, or accompanied with, the rupture of the lining membrane, and this, in fact, is the view of M. Breschet. On this point, however, M. Reynaud joins issue with the above distinguished pathologist for the following reasons.

In pathological investigations it is no uncommon circumstance, says our author, to perceive the internal lining membrane of the heart thickened to a greater or less extent, and of opaque white or yellowish colour. The membrane thus altered is easily detached from the parts which it lines, and, beneath it, instead of the ordinary almost imperceptible cellular tissue, a second thick, whitish, elastic membrane is discovered, capable of being divided into several layers, and resembling, in a great degree, the middle tunic of the arteries. Looking at the results of inflammation in veins, and the mode in which it produces thickening of their coats, M. Reynaud would seem to believe that the same cause, inflammation, operates on the lining membrane of the heart. But this gentleman hunts his analogy a little farther, for he says, that as veins in this thickened state lose their elasticity, and are liable to be dilated by the pressure of the blood, so the lining membrane of the heart being thickened, and likewise deprived of its elasticity, is dilated by the force of its contained blood into an aneurismal pouch.

Such is our author's theory of the disease, and no doubt it is applicable to some cases, though certainly not to all. A ventricle whose lining membrane is thickened, diseased, and inelastic, will probably, nay obviously, be more likely to form these aneurismal sacs than one which is not so affected; still, however, there is no reason why a partial dilatation may not take place in a healthy ventricle from causes of which we are totally ignorant, just as a similar dilatation will occur in a healthy artery. Every one must have witnessed examples of the latter fact, and we shall now put on record what we conceive to be an instance of the former.

On the 5th of last April we witnessed, at St. George's Hospital, the dissection of a young woman, a patient of Dr. Seymour's. The body was not very much attenuated, and omitting, for the present, all other considerations, we shall pass to describe the condition of the heart.

The pericardium verum and reflexum were universally adherent, and the united membranes with the lymph that joined them, were as thick as milled board. Previously to describing what was found in the heart, it is absolutely necessary to notice briefly some points in the anatomy of the organ, which are not dwelt on in the common demonstrations, but are indispensable for the right understanding of the case.

The muscular substance of the left ventricle takes its rise in an abrupt and somewhat angular manner around the ostium ventriculi, from a circular fibrous ring. The muscle of the *ventricle*, as we mentioned before, arises from the lower border of this ligamentous-like ring; and, the muscle of the *auricle*, thin and weak, and not affecting in the least degree the determinate outline of the former, arises in a similar manner from the upper border of the ring, which thus forms the connecting medium between the muscular substance of the auricle and ventricle. The muscle of the auricle, that of the ventricle, and the intervening ring are coated externally by the pericardium verum, which invests all.

The mitral valve is a prolongation and duplicature of the lining membrane of the left auriculo-ventricular cavities, having intervening fibrous substance to give it strength. Between that portion of the membrane lining the wall of the ventricle, and that which forms the outermost layer of the valve, the reflexion of the membrane from one to the other forms a kind of pouch, having at its apex the ligamentous-like ring; on its outside, the muscle of the ventricle; on its inside, the substance of the valve.

Now, in the pouch above described, an aneurism had commenced, and probably, indeed no doubt, was merely a dilatation of this part in its earliest stage, having, on the outside, the muscle of the ventricle; above, the ring; and, on the inside, the valve. As it pushed upwards, however, it rose beyond the origin of the muscle of the *ventricle;* probably threw inwards or expanded over its surface in an attenuated layer the ligamentous ring; and then encroached on the cavity of the *auricle*. Throwing the ring towards its inner side, it would necessarily divert, in the same direction, the muscle of the auricle, which arises from it, and thus would lie between the muscle of the auricle and the pericardium verum.

And this was the state of things in the present case. The pouch was the size of a walnut, and divided into two compartments—it lay in the situation we have sketched out—it was covered towards its inside, above by the muscle and lining membrane of the auricle; below by the mitral valve; and, between the two, by some remains of the ring, of course, though it could not be demonstrated. To the outside it had below, the rounded summit of the muscle of the ventricle; and above this, the inseparably united and thickened layers of the pericardium. It opened into the cavity of the ventricle behind the mitral valve. Its situation was exact'y opposite the septum ventriculorum and origin of the aorta.

The lining membrane of the ventricle which clearly formed the pouch, was apparently ulcerated in two or three places, for coagula were adhering to it, which would probably not have been the case had the membrane remained entire.

Thus, then, supposing, as we must, that some ulceration had occurred, it would obviously constitute false aneurism of the ventricle, although no doubt it was merely a dilatation of the lining membrane in the first instance; in other words, a true aneurism. From the preceding details, (and, however prolix they may appear, they were absolutely necessary to give anything like a scientific or accurate description of the disease,) the following facts are apparent; 1mo. that the lining membrane of the auricle and ventricle was generally sound. 2do. That its dilatation formed the aneurismal pouch. 3tio. That the pouch was neither in the *substance* of the muscle of the ventricle nor that of the auricle, but above the first and to the outside of the second. 4to. That it had no communication with the cavity of the auricle, though it had pushed into, and encroached upon, it; and, 5to. That it was prevented from bursting externally only by the pericardium, which, however, from its thickening, and the intimate union of its two layers, formed a tolerably effectual barrier.

From this, and the other cases upon record, we should be disposed to hazard the following remarks; that the lining membrane of the left ventricle is liable to partial dilatations—that these dilatations may or may not be the result of a diseased condition of the membrane—that they may take place, and most frequently do so, in the muscular substance between the bundles of fibres that compose it; but that they may also occur in another situation, behind the valve, and in the manner we have described—that the pouch may attain some size, without producing any pathognomonic symptoms, unless it should burst, as in the case of General Kyd, and then it is instantly fatal. Thus far we believe that we are borne out by facts, and farther than this it would be madness to venture. Nothing is so dangerous as theorizing;

" ———— Incedimus per ignes
Suppositos cineri doloso, and when facts fail, the writer who has any regard for the *longevity* of his opinions, will do well to stop.

